# Supplementary material for: Potential Therapeutic Effect of Micrornas in Extracellular Vesicles from Mesenchymal Stem Cells against SARS-CoV-2
Source: Cells. 2021 Sep 12;10(9):2393. doi: 10.3390/cells10092393 (PMC8465096; doi:10.3390/cells10092393)
Supplement: Supplementary file 1 [file cells-10-02393-s001.zip › cells-1354393-supplementary.pdf]

cells MDPI

Supplementary information

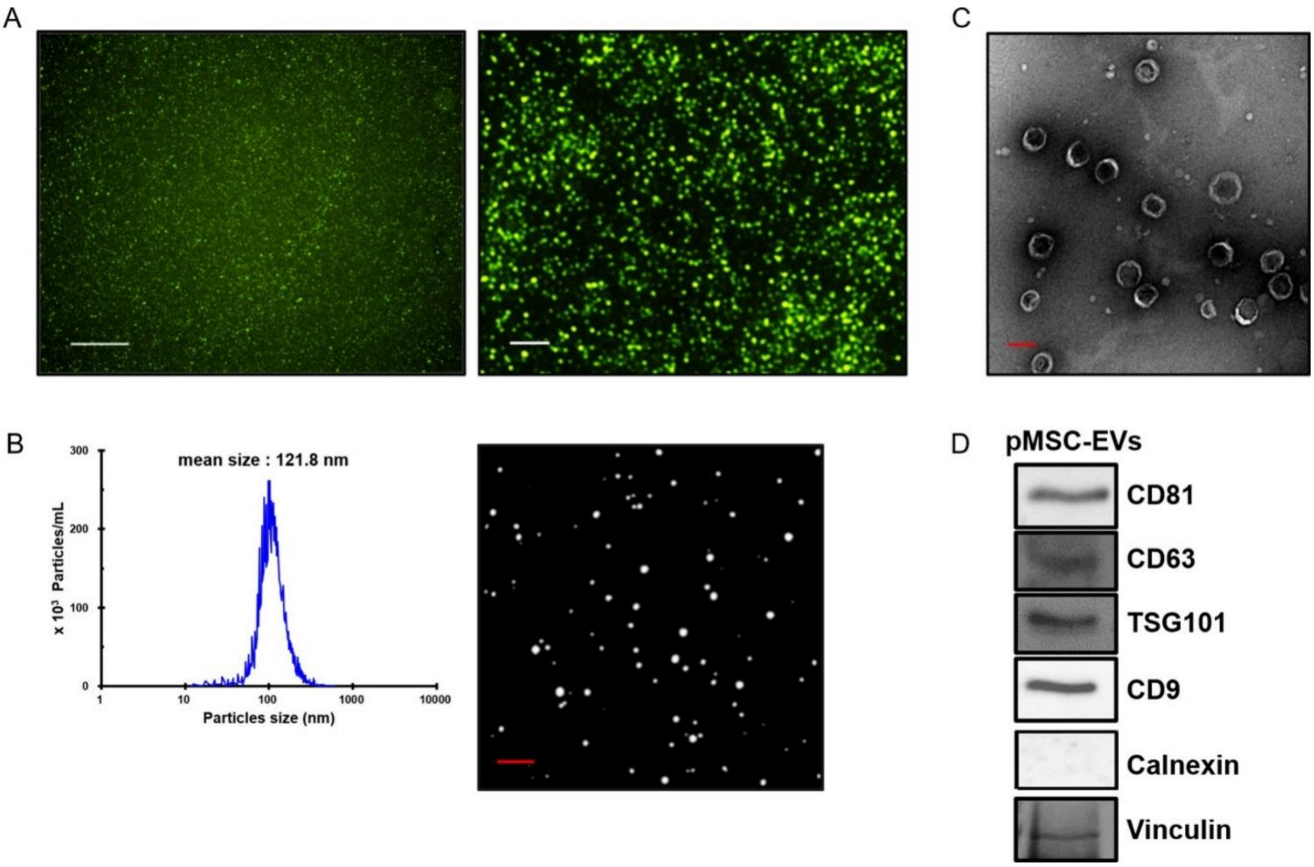

Figure S1. Characterization of human placenta derived mesenchymal stem cells-EVs. (**A**) Fluorescence microscopy images of EVs. EVs were stained for CD63, the common EV marker and Alexa 488 (green). Scale bar: 50 μm (left), 4 μm (right). (**B**) The average size of EVs is 121.8 nm in diameter measured by nanoparticle tracking analysis (NTA) and (**C**) transmission electron microscopy (TEM). Scale bar: 0.1 μm. (**D**) Western blotting detected typical EV markers, including CD81, CD63, TSG101, CD9, and Calnexin, a negative marker.

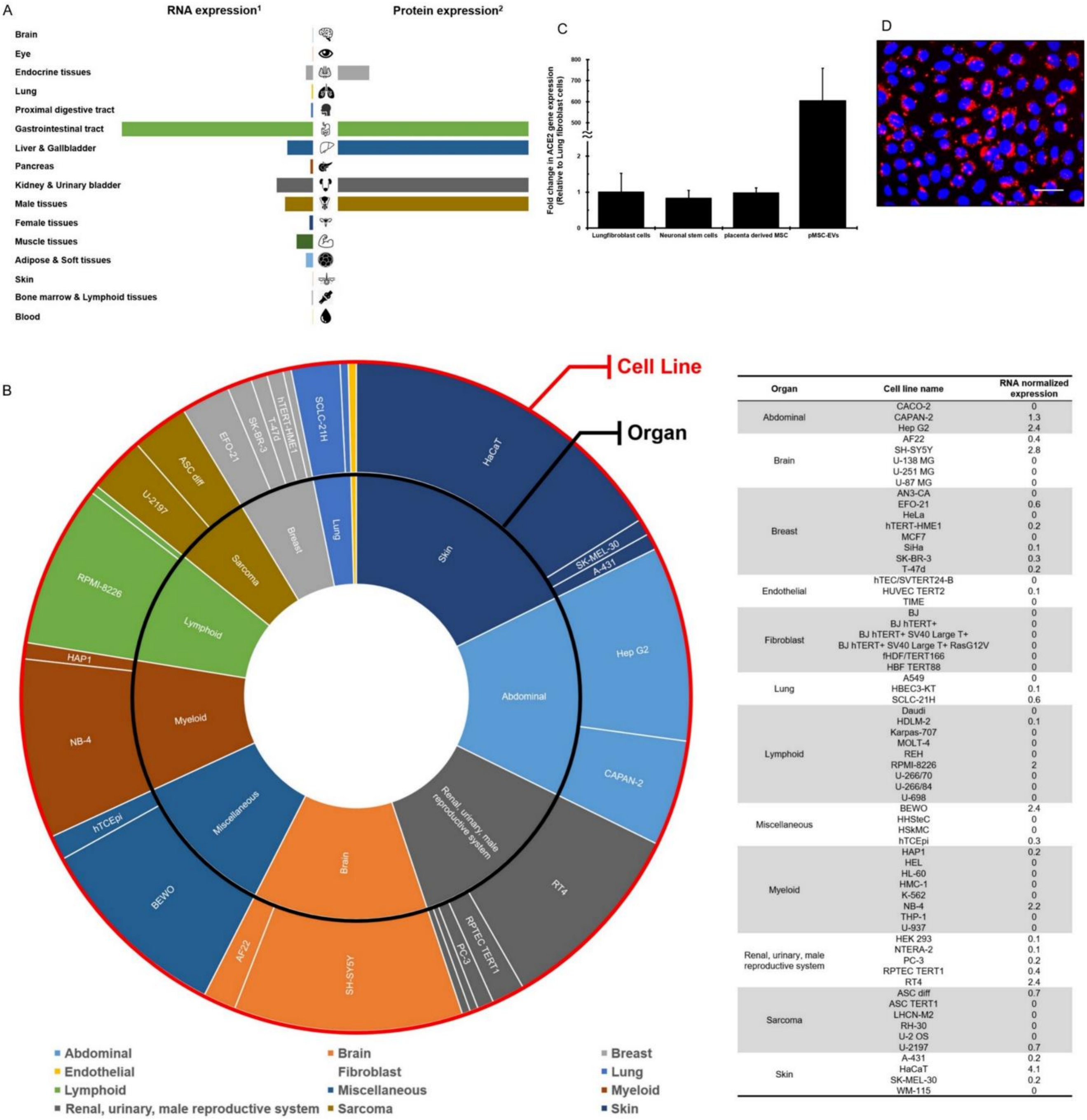

Organ	Cell line name	RNA normalized expression
Abdominal	CACO-2	0
	CAPAN-2	1.3
	Hep G2	2.4
Brain	AF22	0.4
	SH-SY5Y	2.8
	U-138 MG	0
	U-251 MG	0
	U-87 MG	0
Breast	AN3-CA	0
	EFO-21	0.6
	HeLa	0
	hTERT-HME1	0.2
	MCF7	0
	SiHa	0.1
	SK-BR-3	0.3
	T-47d	0.2
Endothelial	hTEC/SVTERT24-B	0
	HUVEC TERT2	0.1
	TIME	0
Fibroblast	BJ	0
	BJ hTERT+	0
	BJ hTERT+ SV40 Large T+	0
	BJ hTERT+ SV40 Large T+ RasG12V	0
	fHDF/TERT166	0
	HBF TERT88	0
Lung	A549	0
	HBEC3-KT	0.1
	SCLC-21H	0.6
Lymphoid	Daudi	0
	HDLM-2	0.1
	Karpas-707	0
	MOLT-4	0
	REH	0
	RPMI-8226	2
	U-266/70	0
	U-266/84	0
	U-698	0
Miscellaneous	BEWO	2.4
	HHSteC	0
	HSkMC	0
	hTCEpi	0.3
Myeloid	HAP1	0.2
	HEL	0
	HL-60	0
	HMC-1	0
	K-562	0
	NB-4	2.2
	THP-1	0
	U-937	0
Renal, urinary, male reproductive system	HEK 293	0.1
	NTERA-2	0.1
	PC-3	0.2
	RPTEC TERT1	0.4
	RT4	2.4
Sarcoma	ASC diff	0.7
	ASC TERT1	0
	LHCN-M2	0
	RH-30	0
	U-2 OS	0
	U-2197	0.7
Skin	A-431	0.2
	HaCaT	4.1
	SK-MEL-30	0.2
	WM-115	0

Figure S2. Organs expressing ACE2 receptors. (**A**) Summarization of ACE2 gene expression level of ACE2 receptor in various human tissues. Data were obtained from Human Protein Atlas (HPA). (1 indicates ACE2 RNA expression measured by RNA sequencing.; 2 indicates ACE2 protein expression measured by in situ hybridization.) (**B**) Summarization of ACE2 expression in human cell lines. Expression data were acquired from HPA. (**C**) Gene expression levels of ACE2 in human lung fibroblasts (LL24), neuronal stem cells (NSCs), pMSCs, and pMSC-EVs. (**D**) Uptake of EVs of PKH26 labelled EVs by Beas-2B cells observed for 24 h after EV treatment.

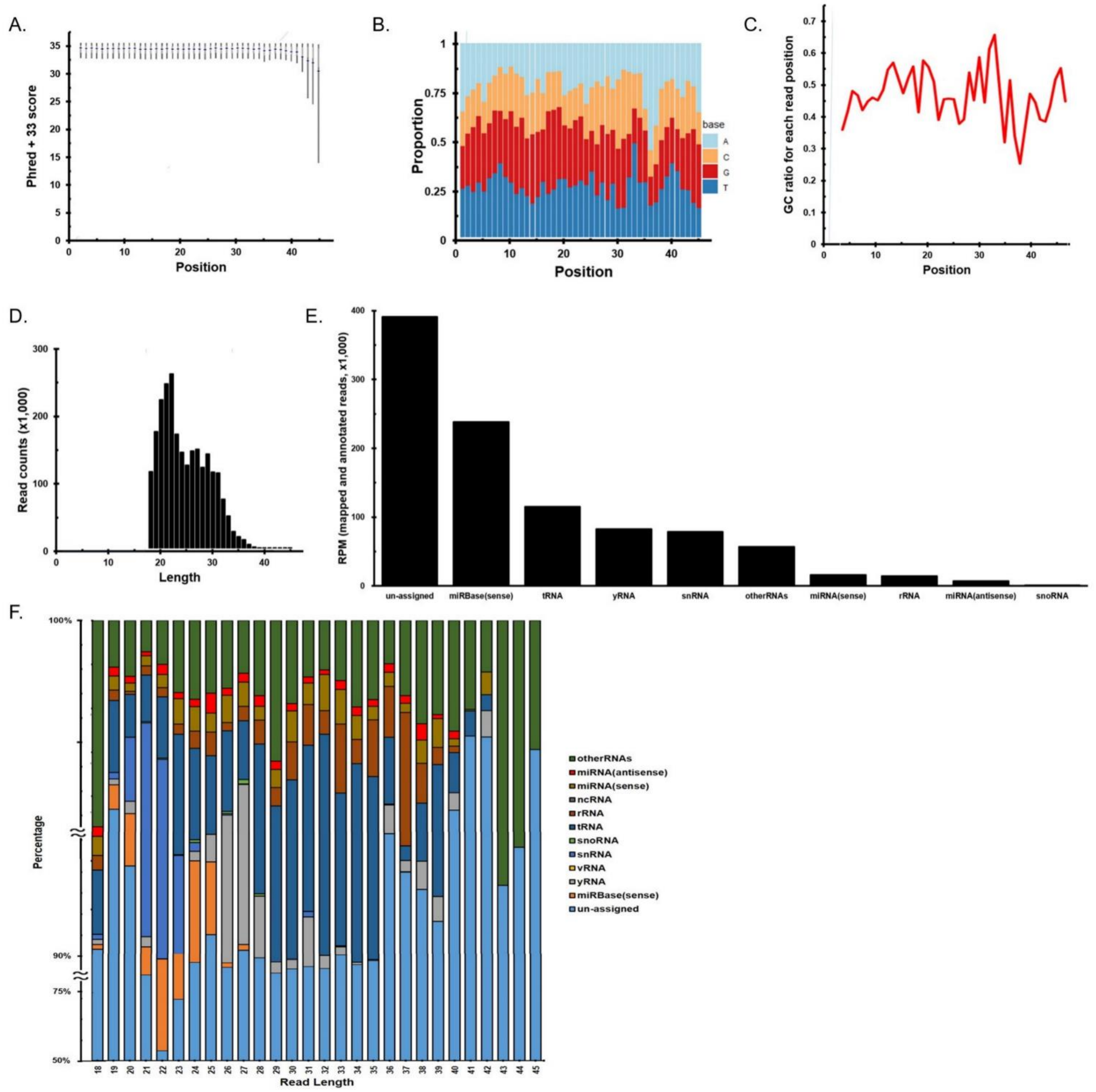

Figure S3. Quality control information for the small RNA sequencing library. (**A**) The Phred + 33 score for each read position. Total number of reads in the library, 24,834,367; average Phred score, 34.8. (**B**) The ratio of A, T, G, and C for each read position. (**C**) The GC ratio for each read position. (**D**) Histogram showing read length distribution. Read length ranged from 18 to 45. Most reads were 18–23 bases in length, corresponding to the size of miRNAs. (**E**) Table showing small miRNA library reads mapped on to the human genome. Unassigned reads were most common, followed by miRNAs, and tRNAs. (**F**) Classification of RNA versus read length. Reads of 20–24 bases, the size of most miRNAs, mapped mainly to miRNAs.

A.

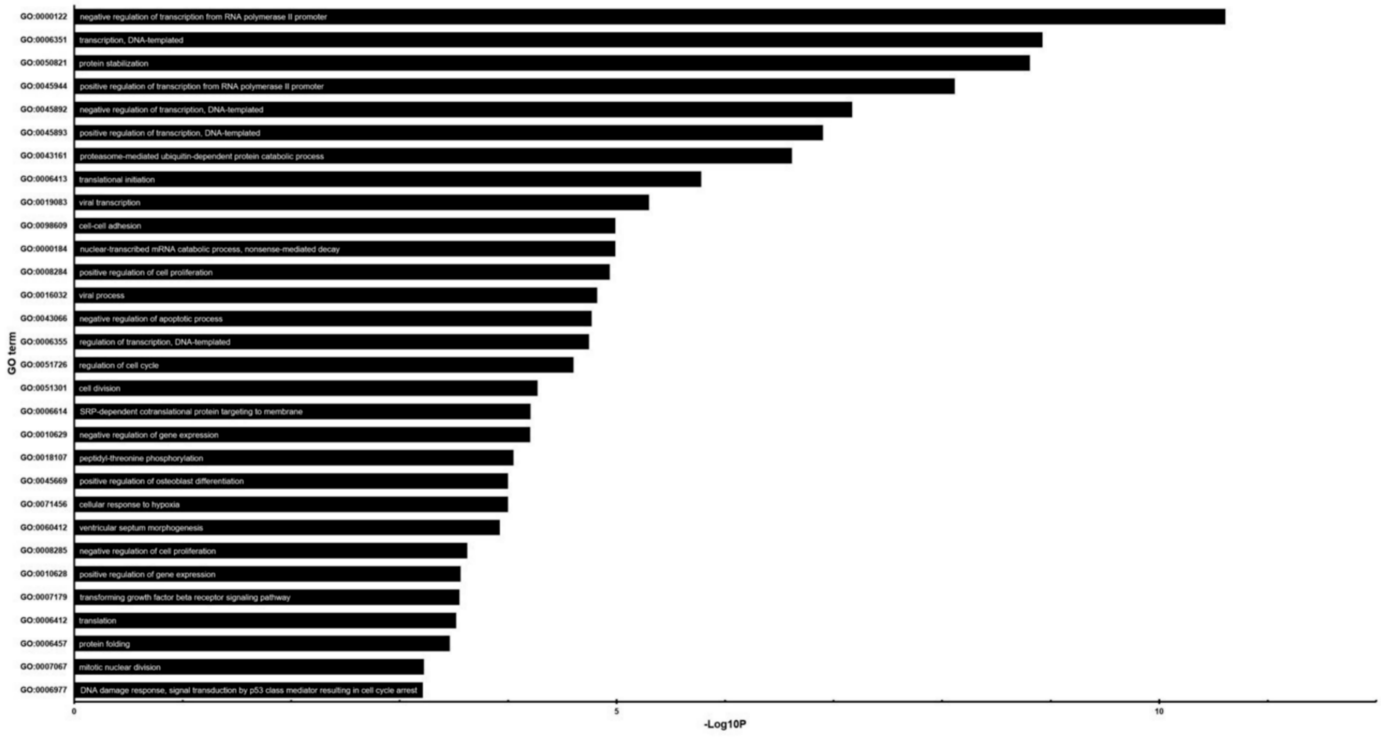
GO term
GO:0000122 negative regulation of transcription from RNA polymerase II promoter
GO:0006351 transcription, DNA-templated
GO:0050821 protein stabilization
GO:0045944 positive regulation of transcription from RNA polymerase II promoter
GO:0045892 negative regulation of transcription, DNA-templated
GO:0045893 positive regulation of transcription, DNA-templated
GO:0043161 proteasome-mediated ubiquitin-dependent protein catabolic process
GO:0006413 translational initiation
GO:0019083 viral transcription
GO:0098609 cell-cell adhesion
GO:0000184 nuclear-transcribed mRNA catabolic process, nonsense-mediated decay
GO:0008284 positive regulation of cell proliferation
GO:0016032 viral process
GO:0043066 negative regulation of apoptotic process
GO:0006355 regulation of transcription, DNA-templated
GO:0051726 regulation of cell cycle
GO:0051301 cell division
GO:0006614 SRP-dependent cotranslational protein targeting to membrane
GO:0010629 negative regulation of gene expression
GO:0018107 peptidyl-threonine phosphorylation
GO:0045669 positive regulation of osteoblast differentiation
GO:0071456 cellular response to hypoxia
GO:0060412 ventricular septum morphogenesis
GO:0008285 negative regulation of cell proliferation
GO:0010628 positive regulation of gene expression
GO:0007179 transforming growth factor beta receptor signaling pathway
GO:0006412 translation
GO:0006457 protein folding
GO:0007067 mitotic nuclear division
GO:0006977 DNA damage response, signal transduction by p53 class mediator resulting in cell cycle arrest
0
5
10
-Log10P

B.

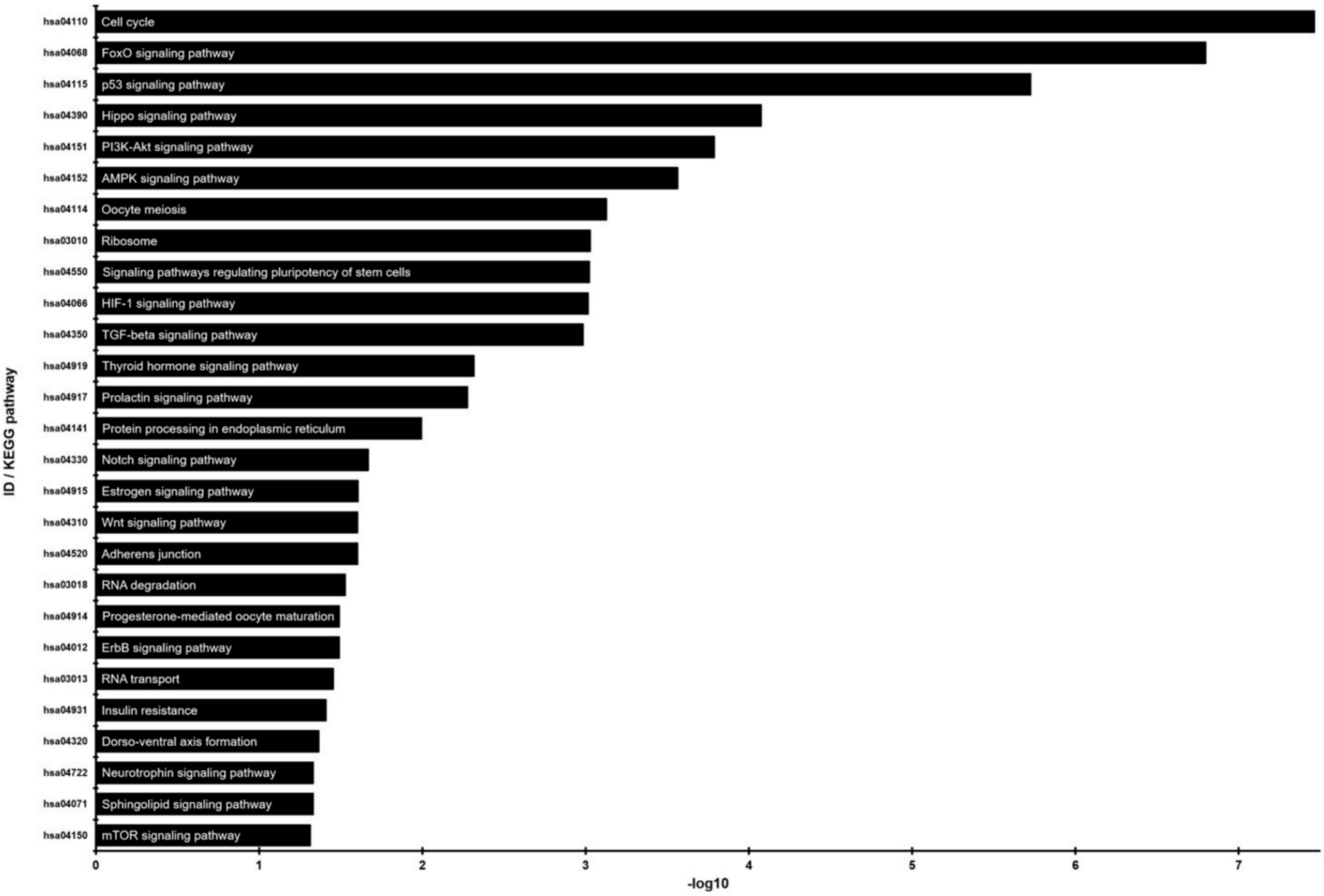
ID / KEGG pathway
hsa04110 Cell cycle
hsa04068 FoxO signaling pathway
hsa04115 p53 signaling pathway
hsa04390 Hippo signaling pathway
hsa04151 PI3K-Akt signaling pathway
hsa04152 AMPK signaling pathway
hsa04114 Oocyte meiosis
hsa03010 Ribosome
hsa04550 Signaling pathways regulating pluripotency of stem cells
hsa04066 HIF-1 signaling pathway
hsa04350 TGF-beta signaling pathway
hsa04919 Thyroid hormone signaling pathway
hsa04917 Prolactin signaling pathway
hsa04141 Protein processing in endoplasmic reticulum
hsa04330 Notch signaling pathway
hsa04915 Estrogen signaling pathway
hsa04310 Wnt signaling pathway
hsa04520 Adherens junction
hsa03018 RNA degradation
hsa04914 Progesterone-mediated oocyte maturation
hsa04012 ErbB signaling pathway
hsa03013 RNA transport
hsa04931 Insulin resistance
hsa04320 Dorso-ventral axis formation
hsa04722 Neurotrophin signaling pathway
hsa04071 Sphingolipid signaling pathway
hsa04150 mTOR signaling pathway
0
1
2
3
4
5
6
7
-log10

C.

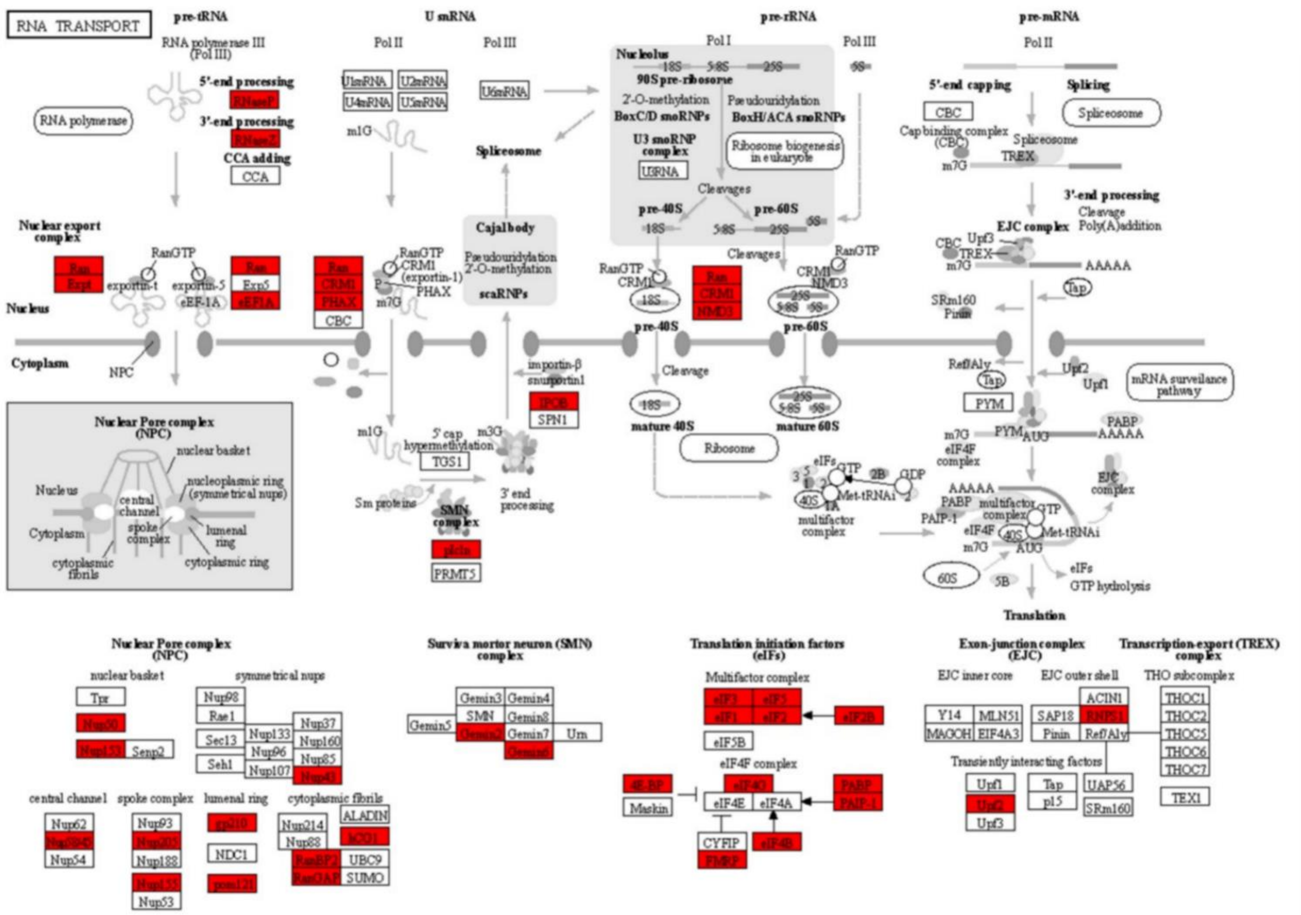
RNA TRANSPORT
pre-tRNA
RNA polymerase III (Pol III)
5'-end processing
3'-end processing
CCA adding
CCA
RNA polymerase
U snRNA
Pol II
Pol III
U1snRNA
U2snRNA
U4snRNA
U5snRNA
U6snRNA
m1G
Spliceosome
pre-rRNA
Pol I
Pol III
Nucleolus
90S pre-ribosome
18S
5.8S
25S
5S
2'-O-methylation
Pseudouridylation
BoxC/D snoRNPs
BoxH/ACA snoRNPs
U3 snoRNP complex
U3RNA
Ribosome biogenesis in eukaryote
Cleavages
pre-40S
pre-60S
pre-mRNA
Pol II
5'-end capping
Splicing
CBC
Spliceosome
Cap binding complex (CBC)
TREX
m7G
3'-end processing
Cleavage
Poly(A)addition
EJC complex
Upf3
AAAAA
Tap
SRm160
Pinin
Nuclear export complex
RanGTP
exportin-t
exportin-5
Exp5
eEF-1A
Nucleus
Cytoplasm
NPC
CRM1 (exportin-1)
PHAX
CBC
Cajal body
Pseudouridylation
2'-O-methylation
scaRNPs
CRM1
NMD3
importin-β
snurportin1
SPN1
5' cap hypermethylation
m3G
TGS1
3' end processing
Sm proteins
SMN complex
PRMT5
Cleavage
mature 40S
mature 60S
Ribosome
eIFs
GTP
2B
GDP
Met-tRNAi
multifactor complex
Ref/Aly
PYM
Upf2
Upf1
mRNA surveillance pathway
PABP
eIF4F complex
EJC complex
AAAAA
PAIP-1
40S
60S
5B
GTP hydrolysis
Translation
Nuclear Pore complex (NPC)
nuclear basket
nucleoplasmic ring (symmetrical nups)
central channel
spoke complex
lumenal ring
cytoplasmic fibrils
cytoplasmic ring
nuclear basket
symmetrical nups
Tpr
Senp2
Nup98
Rae1
Sec13
Seh1
Nup133
Nup96
Nup107
Nup37
Nup160
Nup85
central channel
spoke complex
lumenal ring
cytoplasmic fibrils
Nup62
Nup54
Nup93
Nup188
Nup53
NDC1
Nup214
Nup88
ALADIN
UBC9
SUMO
Surviva mortor neuron (SMN) complex
Gemin3
Gemin4
SMN
Gemin8
Gemin5
Gemin7
Unr
Translation initiation factors (eIFs)
Multifactor complex
eIF5B
eIF4F complex
Maskin
eIF4E
eIF4A
CYFIP
Exon-junction complex (EJC)
EJC inner core
EJC outer shell
Y14
MLN51
MAGOH
EIF4A3
ACIN1
SAP18
Pinin
Ref/Aly
Transiently interacting factors
Upf1
Upf3
Tap
p15
UAP56
SRm160
Transcription-export (TREX) complex
THO subcomplex
THOC1
THOC2
THOC5
THOC6
THOC7
TEX1

D.

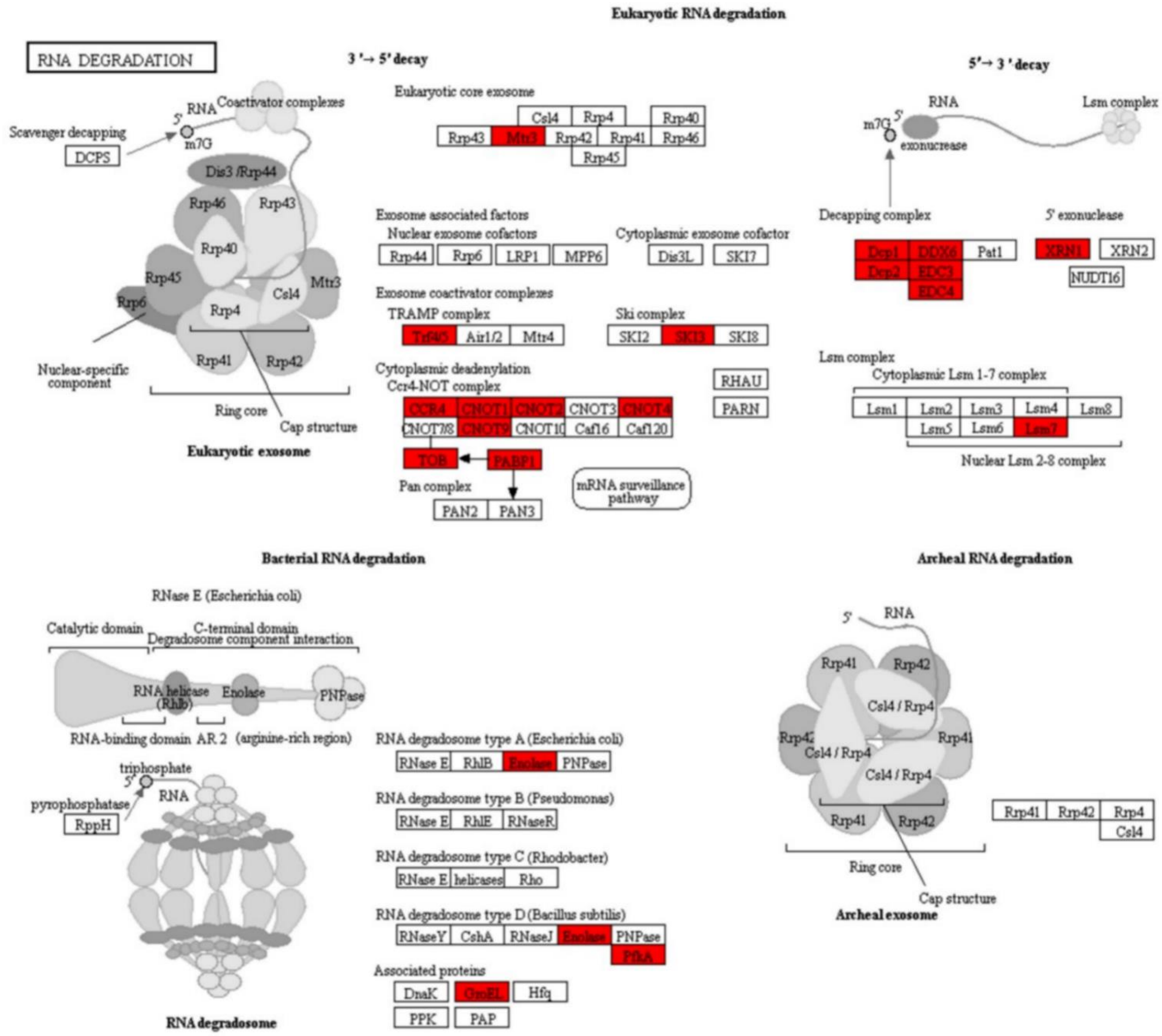
RNA DEGRADATION
Eukaryotic RNA degradation
3 '→ 5' decay
5'→ 3 ' decay
Eukaryotic core exosome
Csl4
Rrp4
Rrp40
Rrp43
Mtr3
Rrp42
Rrp41
Rrp46
Rrp45
Scavenger decapping
DCPS
RNA
Coactivator complexes
m7G
Dis3 /Rrp44
Rrp6
Nuclear-specific component
Ring core
Cap structure
Eukaryotic exosome
Exosome associated factors
Nuclear exosome cofactors
Rrp44
LRP1
MPP6
Cytoplasmic exosome cofactor
Dis3L
SKI7
Exosome coactivator complexes
TRAMP complex
Trf4/5
Air1/2
Mtr4
Ski complex
SKI2
SKI3
SKI8
Cytoplasmic deadenylation
Ccr4-NOT complex
CCR4
CNOT1
CNOT2
CNOT3
CNOT4
CNOT7/8
CNOT9
CNOT10
Caf16
Caf120
TOB
PABP1
Pan complex
PAN2
PAN3
mRNA surveillance pathway
RHAU
PARN
exonucrease
Lsm complex
Decapping complex
Dcp1
DDX6
Pat1
Dcp2
EDC3
EDC4
5' exonuclease
XRN1
XRN2
NUDT16
Cytoplasmic Lsm 1-7 complex
Lsm1
Lsm2
Lsm3
Lsm4
Lsm8
Lsm5
Lsm6
Lsm7
Nuclear Lsm 2-8 complex
Bacterial RNA degradation
RNase E (Escherichia coli)
Catalytic domain
C-terminal domain
Degradosome component interaction
RNA helicase (Rhlb)
Enolase
PNPase
RNA-binding domain
AR 2
(arginine-rich region)
triphosphate
pyrophosphatase
RppH
RNA degradosome
RNA degradosome type A (Escherichia coli)
RNase E
RhlB
Enolase
PNPase
RNA degradosome type B (Pseudomonas)
RhlE
RNaseR
RNA degradosome type C (Rhodobacter)
helicases
Rho
RNA degradosome type D (Bacillus subtilis)
RNaseY
CshA
RNaseJ
PfkA
Associated proteins
DnaK
GroEL
Hfq
PPK
PAP
Archeal RNA degradation
Csl4 / Rrp4
Archeal exosome

E.

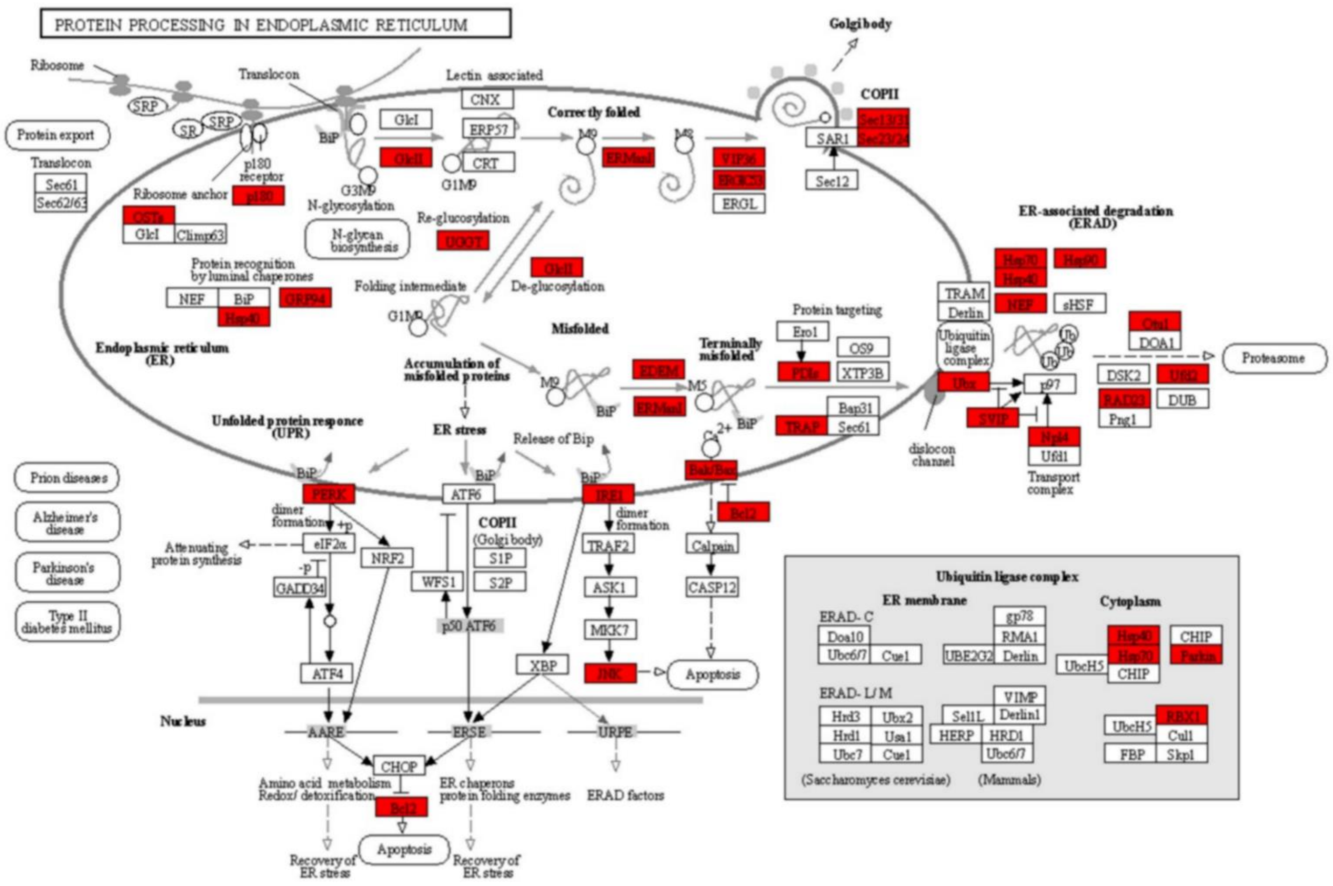
PROTEIN PROCESSING IN ENDOPLASMIC RETICULUM
Ribosome
Translocon
SRP
SR
Protein export
Translocon
Sec61
Sec62/63
p180 receptor
Ribosome anchor
BiP
G3M9
N-glycosylation
N-glycan biosynthesis
Lectin associated
CNX
ERP57
CRT
G1M9
GlcI
Correctly folded
M9
M8
ERGL
Sec12
SAR1
COPII
Golgi body
Re-glucosylation
De-glucosylation
Folding intermediate
G1M9
Protein recognition by luminal chaperones
NEF
BiP
GlcI
Climp63
Endoplasmic reticulum (ER)
Misfolded
Accumulation of misfolded proteins
M9
BiP
Terminally misfolded
M5
Protein targeting
Ero1
OS9
XTP3B
Bap31
Sec61
ER-associated degradation (ERAD)
TRAM
Derlin
sHSF
Ubiquitin ligase complex
p97
Ub
DSK2
DOA1
DUB
Png1
Proteasome
Ufd1
Transport complex
dislocon channel
Unfolded protein responce (UPR)
ER stress
Release of Bip
Ca^{2+}
BiP
PERK
ATF6
IRE1
dimer formation
+p
-p
eIF2α
Attenuating protein synthesis
GADD34
NRF2
ATF4
WFS1
COPII (Golgi body)
S1P
S2P
p50 ATF6
TRAF2
ASK1
MKK7
XBP
Calpain
CASP12
Apoptosis
Prion diseases
Alzheimer's disease
Parkinson's disease
Type II diabetes mellitus
Nucleus
AARE
ERSE
URPE
CHOP
Amino acid metabolism Redox/ detoxification
ER chaperons protein folding enzymes
ERAD factors
Recovery of ER stress
Apoptosis
Recovery of ER stress
Ubiquitin ligase complex
ER membrane
Cytoplasm
ERAD- C
Doa10
Ubc6/7
Cue1
gp78
RMA1
UBE2G2
Derlin
ERAD- L/ M
Hrd3
Ubx2
Hrd1
Usa1
Ubc7
Cue1
VIMP
Sel1L
Derlin1
HERP
HRD1
Ubc6/7
CHIP
UbcH5
CHIP
UbcH5
Cul1
FBP
Skp1
(Saccharomyces cerevisiae)
(Mammals)

F.

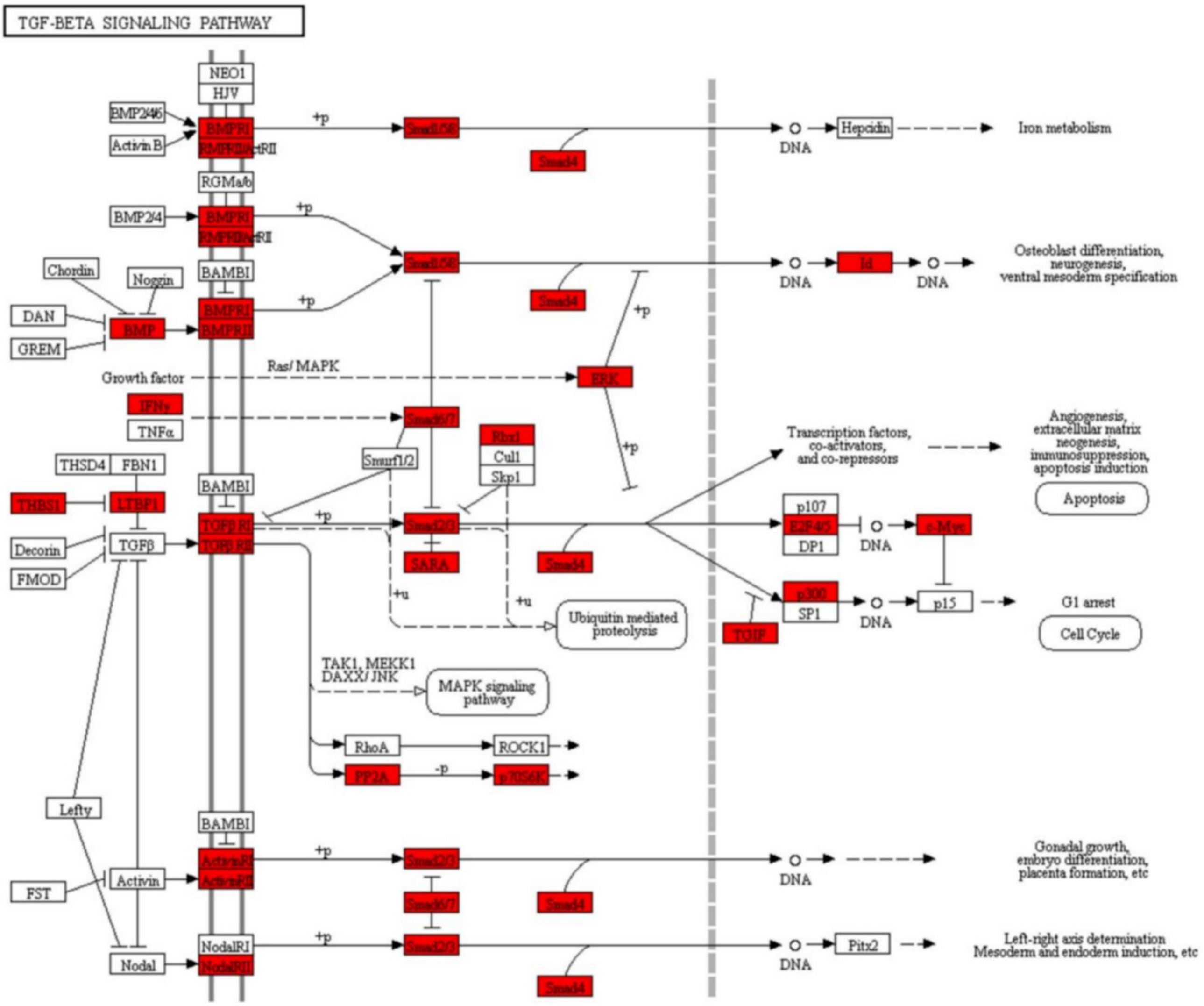

Figure S4. Function of mRNAs targeted by the five miRNAs. (**A**) Statistical enrichment analysis of GO terms for the target genes of the miRNAs. The plot shows the top 30 GO terms. The x axis shows minus log10 transformed adjusted p-values and the y axis shows the GO terms. (**B**) Statistical enrichment analysis of 83 inflammatory associated genes in KEGG pathways. The pathway showing adjusted p-values < 0.05 is presented in the plot. The x axis shows minus log10 transformed and adjusted p-values, and the y axis shows the names of the KEGG pathway i.e., RNA transport (**C**), RNA degradation (**D**), protein processing in endoplasmic reticulum (**E**), and TGF-BETA signaling pathway (**F**). The red box in each pathway indicates the gene targeted directly by miRNAs.

A.

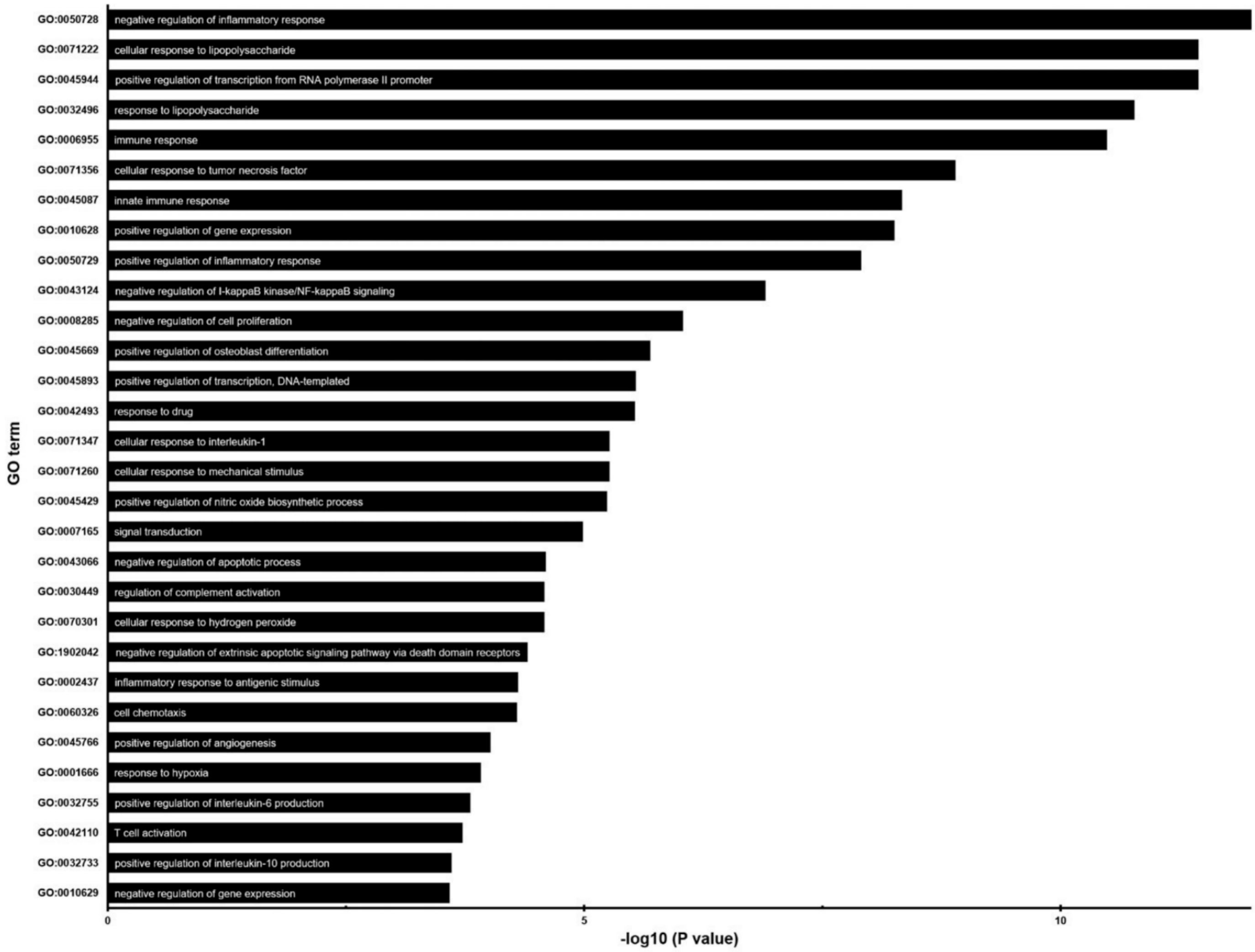
GO:0050728 negative regulation of inflammatory response
GO:0071222 cellular response to lipopolysaccharide
GO:0045944 positive regulation of transcription from RNA polymerase II promoter
GO:0032496 response to lipopolysaccharide
GO:0006955 immune response
GO:0071356 cellular response to tumor necrosis factor
GO:0045087 innate immune response
GO:0010628 positive regulation of gene expression
GO:0050729 positive regulation of inflammatory response
GO:0043124 negative regulation of I-kappaB kinase/NF-kappaB signaling
GO:0008285 negative regulation of cell proliferation
GO:0045669 positive regulation of osteoblast differentiation
GO:0045893 positive regulation of transcription, DNA-templated
GO:0042493 response to drug
GO:0071347 cellular response to interleukin-1
GO:0071260 cellular response to mechanical stimulus
GO:0045429 positive regulation of nitric oxide biosynthetic process
GO:0007165 signal transduction
GO:0043066 negative regulation of apoptotic process
GO:0030449 regulation of complement activation
GO:0070301 cellular response to hydrogen peroxide
GO:1902042 negative regulation of extrinsic apoptotic signaling pathway via death domain receptors
GO:0002437 inflammatory response to antigenic stimulus
GO:0060326 cell chemotaxis
GO:0045766 positive regulation of angiogenesis
GO:0001666 response to hypoxia
GO:0032755 positive regulation of interleukin-6 production
GO:0042110 T cell activation
GO:0032733 positive regulation of interleukin-10 production
GO:0010629 negative regulation of gene expression
GO term
0
5
10
-log10 (P value)

B.

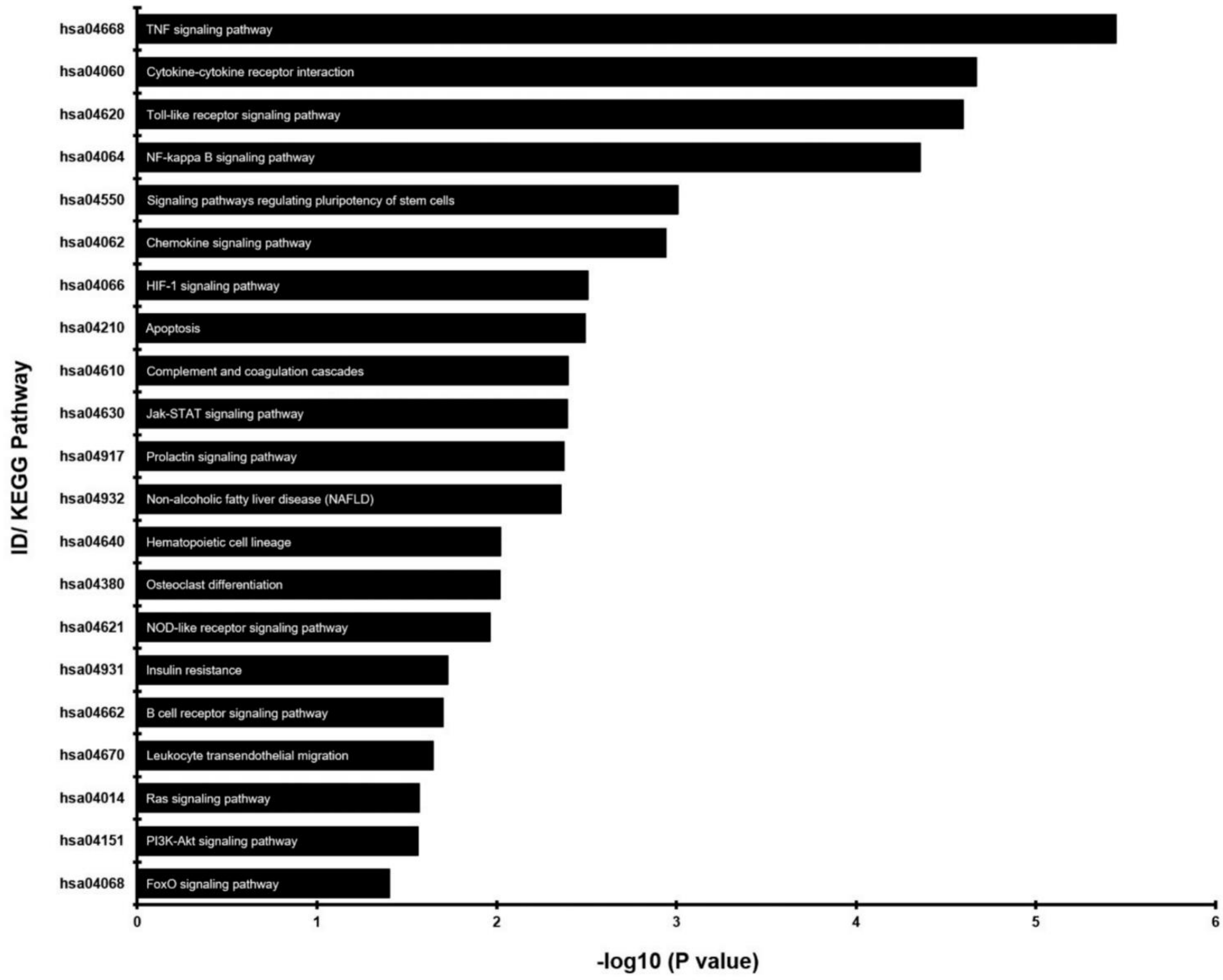
ID/ KEGG Pathway
hsa04668 TNF signaling pathway
hsa04060 Cytokine-cytokine receptor interaction
hsa04620 Toll-like receptor signaling pathway
hsa04064 NF-kappa B signaling pathway
hsa04550 Signaling pathways regulating pluripotency of stem cells
hsa04062 Chemokine signaling pathway
hsa04066 HIF-1 signaling pathway
hsa04210 Apoptosis
hsa04610 Complement and coagulation cascades
hsa04630 Jak-STAT signaling pathway
hsa04917 Prolactin signaling pathway
hsa04932 Non-alcoholic fatty liver disease (NAFLD)
hsa04640 Hematopoietic cell lineage
hsa04380 Osteoclast differentiation
hsa04621 NOD-like receptor signaling pathway
hsa04931 Insulin resistance
hsa04662 B cell receptor signaling pathway
hsa04670 Leukocyte transendothelial migration
hsa04014 Ras signaling pathway
hsa04151 PI3K-Akt signaling pathway
hsa04068 FoxO signaling pathway
0
1
2
3
4
5
6
-log10 (P value)

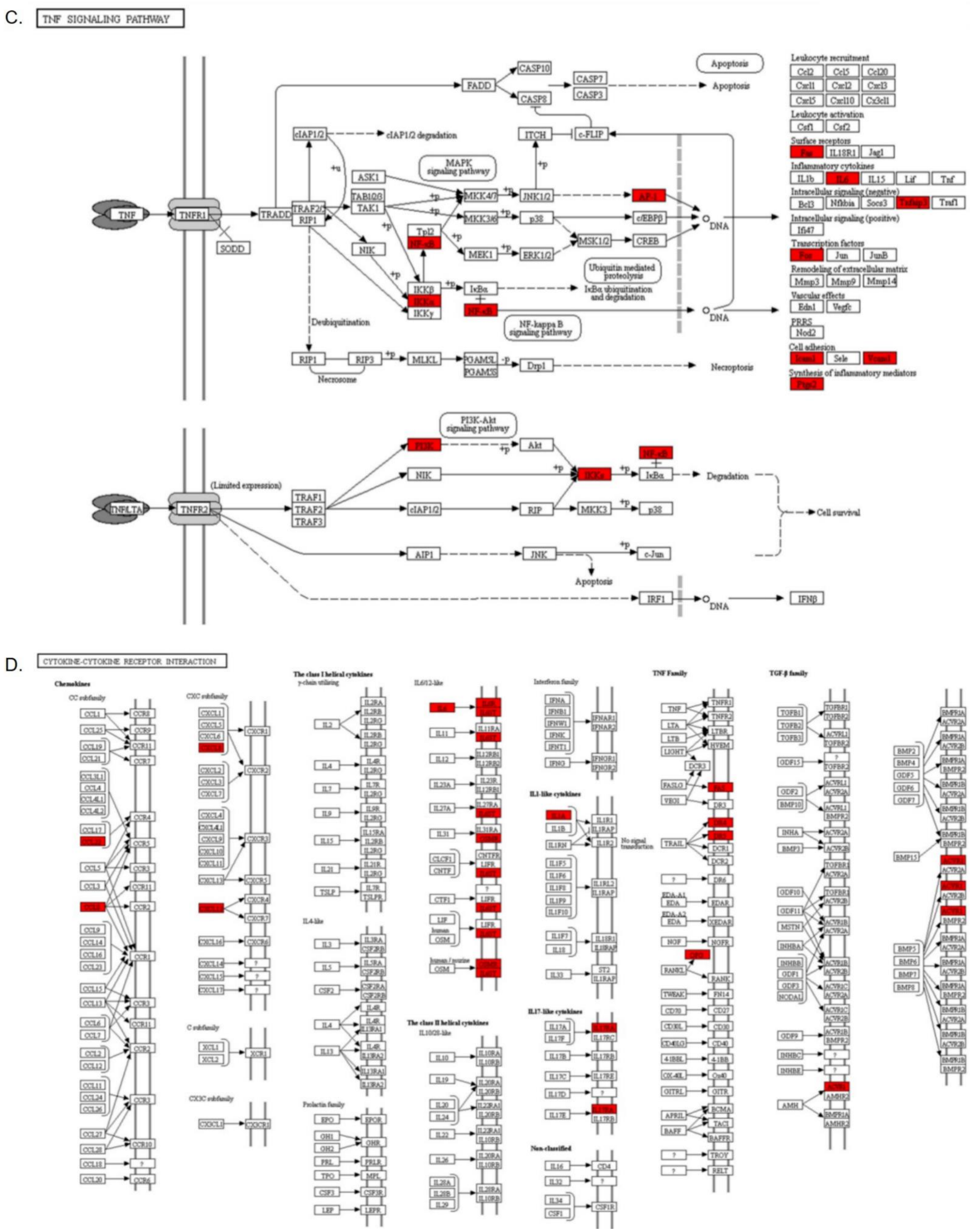
C.
TNF SIGNALING PATHWAY
TNF
TNFR1
SODD
TRADD
TRAF2/5
RIP1
cIAP1/2
cIAP1/2 degradation
+u
FADD
CASP10
CASP8
CASP7
CASP3
Apoptosis
ITCH
c-FLIP
ASK1
TAB1/2/3
TAK1
MAPK signaling pathway
MKK4/7
MKK3/6
JNK1/2
p38
Tpl2
MEK1
ERK1/2
MSK1/2
c/EBPβ
CREB
DNA
NIK
IKKβ
IKKγ
IκBα
Ubiquitin mediated proteolysis
IκBα ubiquitination and degradation
NF-kappa B signaling pathway
Deubiquitination
RIP1
RIP3
MLKL
PGAM5L
PGAM5S
Drp1
Necrosome
Necroptosis
+p
Leukocyte recruitment
Ccl2
Ccl5
Ccl20
Cxcl1
Cxcl2
Cxcl3
Cxcl5
Cxcl10
Cx3cl1
Leukocyte activation
Csf1
Csf2
Surface receptors
IL18R1
Jag1
Inflammatory cytokines
IL1b
IL15
Lif
Tnf
Intracellular signaling (negative)
Bcl3
Nfkbia
Socs3
Traf1
Intracellular signaling (positive)
Ifi47
Transcription factors
Jun
JunB
Remodeling of extracellular matrix
Mmp3
Mmp9
Mmp14
Vascular effects
Edn1
Vegfc
PRRS
Nod2
Cell adhesion
Sele
Synthesis of inflammatory mediators
PI3K-Akt signaling pathway
Akt
NF-κB
IκBα
Degradation
TNF/LTA
TNFR2
(Limited expression)
TRAF1
TRAF2
TRAF3
NIK
cIAP1/2
RIP
MKK3
p38
Cell survival
AIP1
JNK
c-Jun
Apoptosis
IRF1
DNA
IFNβ
D.
CYTOKINE-CYTOKINE RECEPTOR INTERACTION
Chemokines
CC subfamily
CXC subfamily
C subfamily
CX3C subfamily
The class I helical cytokines
IL6/12-like
IL4-like
Prolactin family
Interferon family
IL1-like cytokines
IL17-like cytokines
The class II helical cytokines
IL10/28-like
Non-classified
TNF Family
TGF-β family

E.

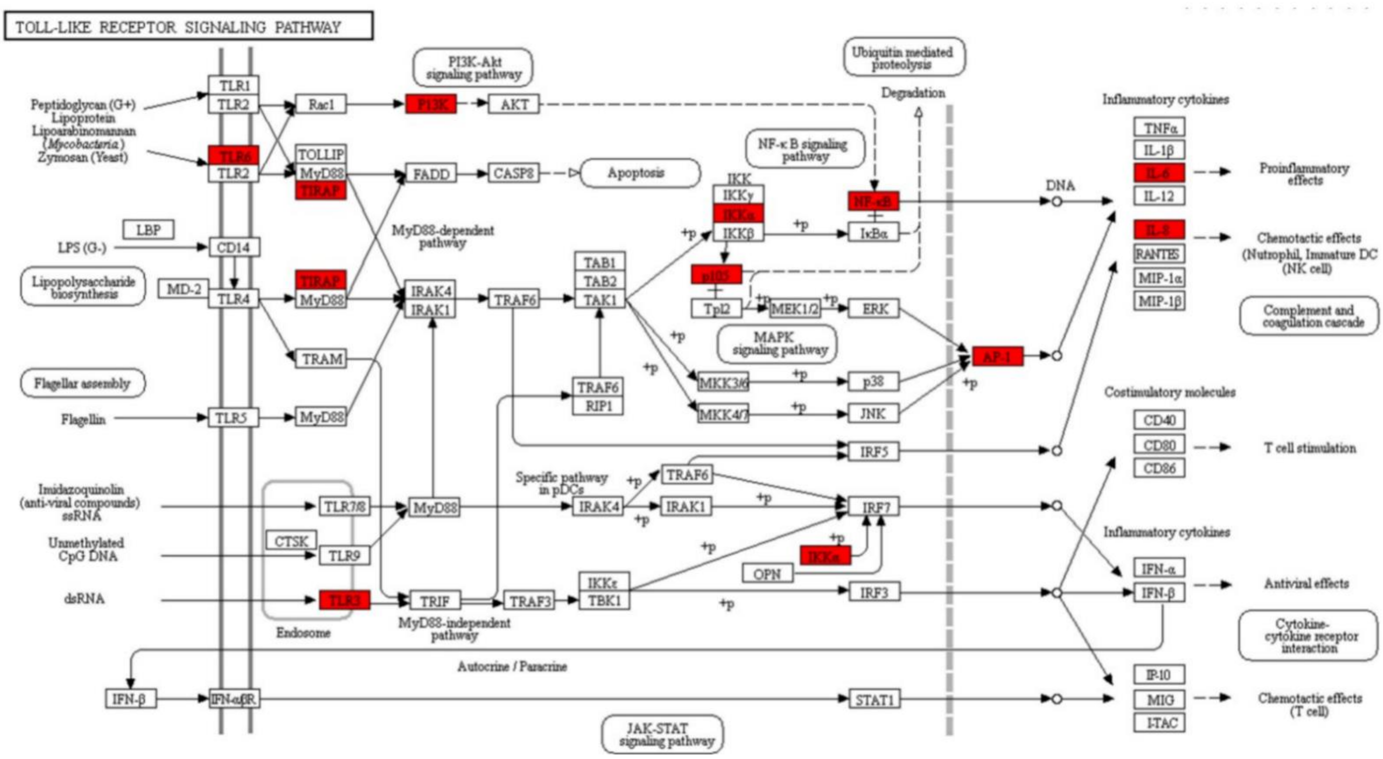
TOLL-LIKE RECEPTOR SIGNALING PATHWAY
PI3K-Akt signaling pathway
Ubiquitin mediated proteolysis
Degradation
Peptidoglycan (G+)
Lipoprotein
Lipoarabinomannan (Mycobacteria)
Zymosan (Yeast)
TLR1
TLR2
TLR6
Rac1
PI3K
AKT
TOLLIP
MyD88
TIRAP
FADD
CASP8
Apoptosis
NF-κB signaling pathway
IKK
IKKγ
IKKα
IKKβ
NF-κB
IκBα
DNA
Inflammatory cytokines
TNFα
IL-1β
IL-6
IL-12
Proinflammatory effects
IL-8
RANTES
MIP-1α
MIP-1β
Chemotactic effects (Nutrophil, Immature DC (NK cell)
Complement and coagulation cascade
LBP
LPS (G-)
CD14
MD-2
TLR4
Lipopolysaccharide biosynthesis
MyD88-dependent pathway
IRAK4
IRAK1
TRAF6
TAB1
TAB2
TAK1
p105
Tpl2
MEK1/2
ERK
MAPK signaling pathway
AP-1
TRAM
Flagellar assembly
Flagellin
TLR5
RIP1
MKK3/6
MKK4/7
p38
JNK
Costimulatory molecules
CD40
CD80
CD86
T cell stimulation
IRF5
Imidazoquinolin (anti-viral compounds) ssRNA
TLR7/8
Specific pathway in pDCs
IRF7
Unmethylated CpG DNA
CTSK
TLR9
OPN
Inflammatory cytokines
IFN-α
IFN-β
Antiviral effects
dsRNA
TLR3
TRIF
TRAF3
IKKε
TBK1
IRF3
Endosome
MyD88-independent pathway
Cytokine-cytokine receptor interaction
Autocrine / Paracrine
IFN-αβR
STAT1
IP-10
MIG
I-TAC
Chemotactic effects (T cell)
JAK-STAT signaling pathway
+p

F.

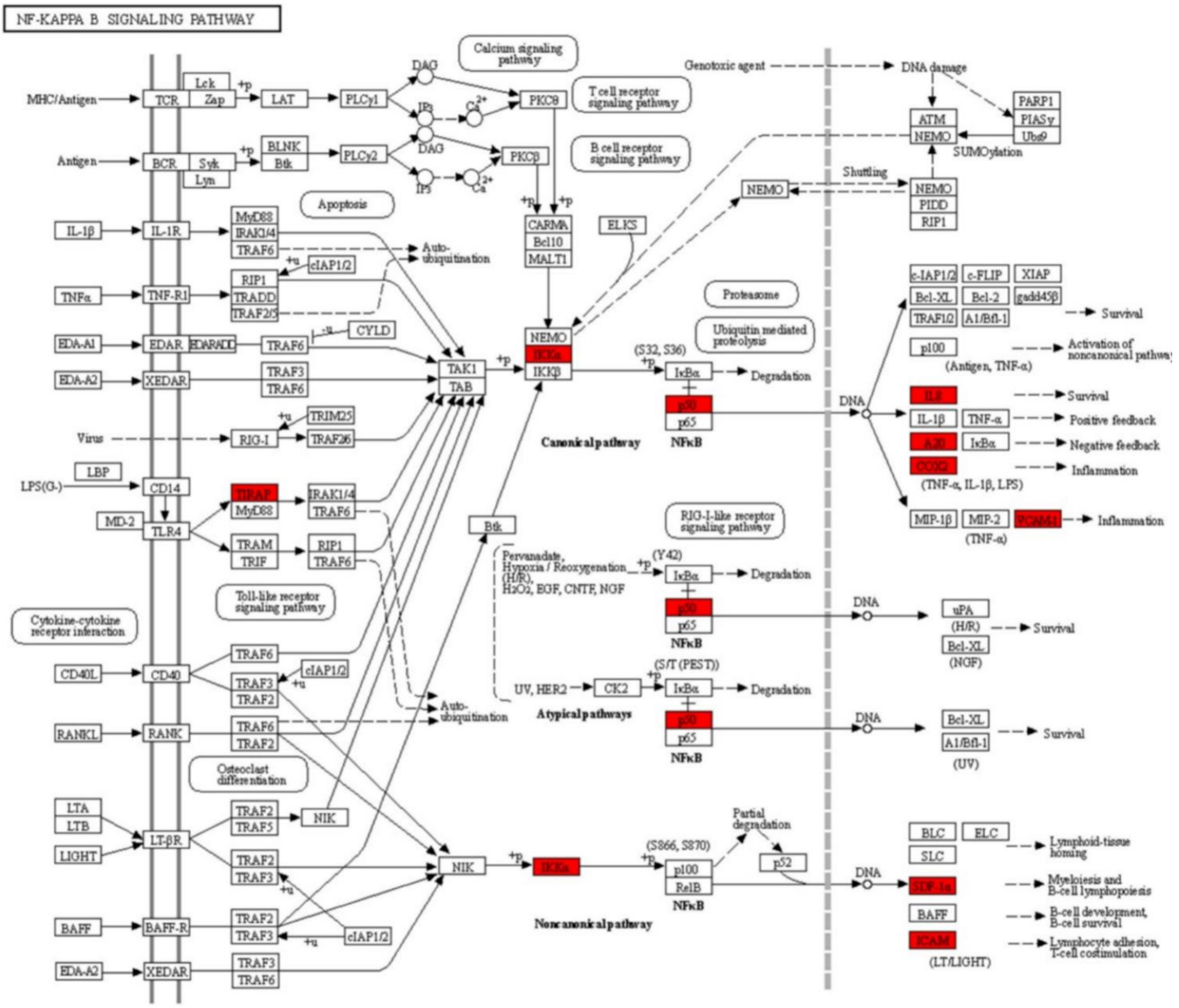
NF-KAPPA B SIGNALING PATHWAY
Calcium signaling pathway
T cell receptor signaling pathway
B cell receptor signaling pathway
Apoptosis
Proteasome
Ubiquitin mediated proteolysis
Toll-like receptor signaling pathway
RIG-I-like receptor signaling pathway
Cytokine-cytokine receptor interaction
Osteoclast differentiation
MHC/Antigen
Antigen
TCR
BCR
Lck
Zap
Syk
Lyn
LAT
BLNK
Btk
PLCγ1
PLCγ2
DAG
IP3
Ca2+
PKCθ
PKCβ
CARMA
Bcl10
MALT1
ELKS
IL-1β
IL-1R
MyD88
IRAK1/4
TRAF6
Auto-ubiquitination
cIAP1/2
TNFα
TNF-R1
RIP1
TRADD
TRAF2/5
EDA-A1
EDAR
EDARADD
CYLD
EDA-A2
XEDAR
TRAF3
TAK1
TAB
NEMO
IKKα
IKKβ
Virus
RIG-I
TRIM25
TRAF2/6
LBP
LPS(G-)
CD14
MD-2
TLR4
TIRAP
TRAM
TRIF
Btk
CD40L
CD40
RANKL
RANK
TRAF2
LTA
LTB
LIGHT
LT-βR
TRAF5
NIK
BAFF
BAFF-R
Genotoxic agent
DNA damage
ATM
PARP1
PIASy
Ubc9
SUMOylation
Shuttling
PIDD
(S32, S36)
IκBα
Degradation
p50
p65
NFκB
Canonical pathway
DNA
c-IAP1/2
c-FLIP
XIAP
Bcl-XL
Bcl-2
gadd45β
TRAF1/2
A1/Bfl-1
Survival
p100
Activation of noncanonical pathway
(Antigen, TNF-α)
IL8
IL-1β
TNF-α
Positive feedback
A20
Negative feedback
COX2
Inflammation
(TNF-α, IL-1β, LPS)
MIP-1β
MIP-2
ICAM1
(TNF-α)
Pervanadate, Hypoxia / Reoxygenation (H/R), H2O2, EGF, CNTF, NGF
(Y42)
uPA
(H/R)
Bcl-XL
(NGF)
UV, HER2
CK2
(S/T (PEST))
Atypical pathways
A1/Bfl-1
(UV)
Partial degradation
(S866, S870)
p52
RelB
Noncanonical pathway
BLC
ELC
SLC
SDF-1α
ICAM
Lymphoid-tissue homing
Myeloiesis and B-cell lymphopoiesis
B-cell development, B-cell survival
Lymphocyte adhesion, T-cell costimulation
(LT/LIGHT)

G.

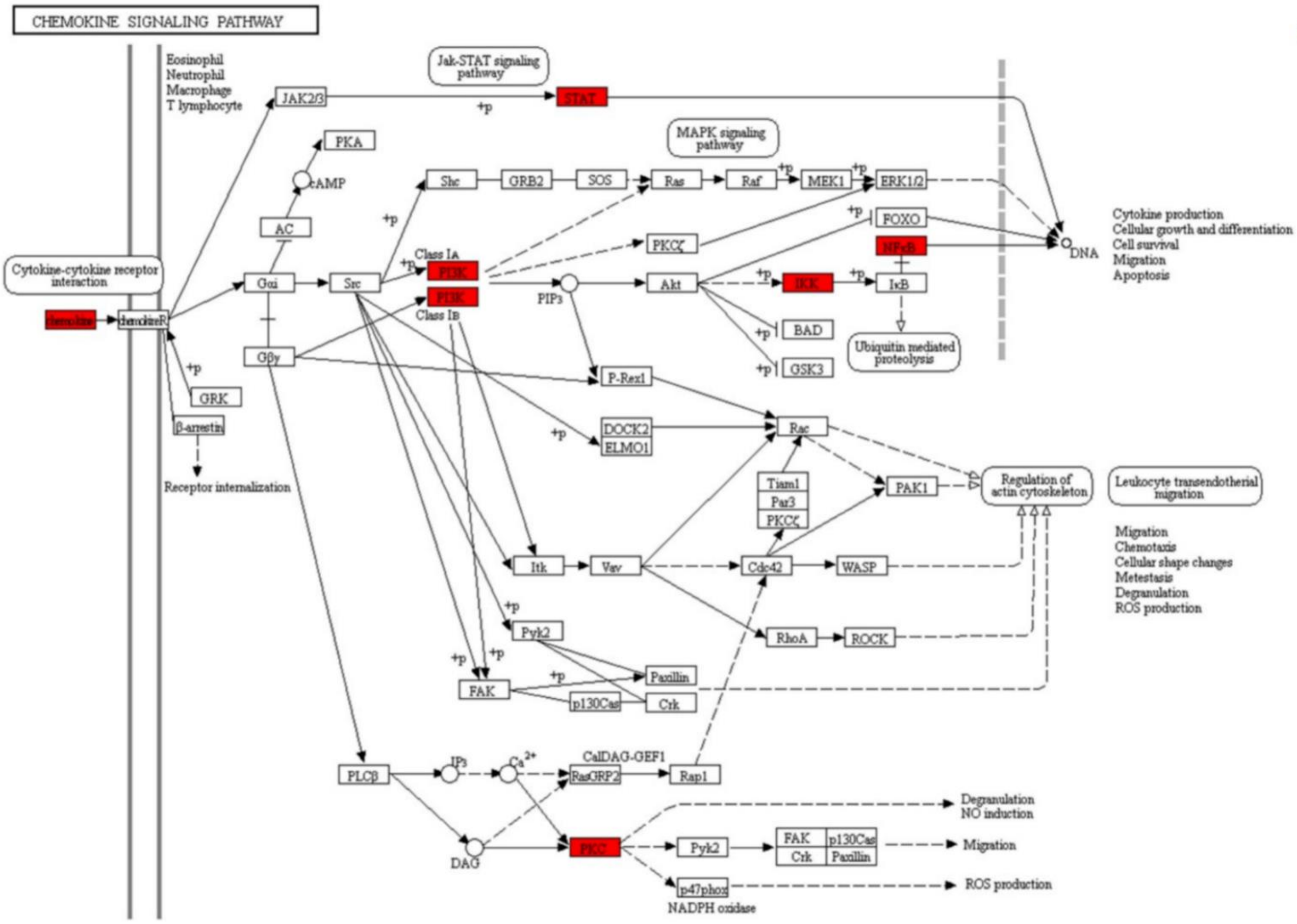

Figure S5. Significant inflammatory KEGG pathways regulating by the targets of the five miRNAs. (**A**) Statistical enrichment analysis of GO terms for 83 inflammatory associated genes. The plot shows the top 30 GO terms. The X axis shows minus log10 transformed and adjusted p-values and the y axis shows the GO terms. (**B**) Significant pathways involving the 83 genes targeted by the five miRNAs. The 83 genes are predicted to be involved in inflammatory responses through pathways such as TNF signaling (**C**), Toll-like receptor signaling (**D**), cytokine-cytokine interactions (**E**), NF-kAPPA B signaling (**F**), and chemokine signaling (**G**). The red box in each pathway indicates the gene targeted directly by the miRNAs.

Table S1. The expression levels of 84 common miRNAs present in EVs derived from eight MSCs cultured under various conditions and six PD-EVs.

miRNA	ID	MSC1-EVs	MSC2-EVs	MSC3-EVs	MSC4-EVs	MSC5-EVs	MSC6-EVs	MSC7-EVs	MSC8-EVs	PD1-EVs	PD2-EVs	PD3-EVs	PD4-EVs	PD5-EVs	PD6-EVs
hsa-let-7a-5p	MIMAT0000062	94794.5	174629.0	46080.8	143994.2	150493.2	149839.8	85700.1	143439.6	144317.8	61717.9	162406.0	183726.9	107008.0	44679.7
hsa-let-7b-5p	MIMAT0000063	24148.8	27689.1	27639.8	36074.7	40177.1	42825.4	30255.1	41274.1	6056.7	22314.1	67574.5	66459.6	60121.6	126851.1
hsa-let-7c-5p	MIMAT0000064	12691.4	15227.9	7272.3	2232.5	1315.5	1350.5	1631.8	1597.7	292.5	5093.9	4038.8	4228.7	22756.9	2550.9
hsa-let-7d-3p	MIMAT0004484	2471.4	2467.9	2261.5	1311.6	3020.3	4473.8	5158.8	4006.1	101.5	475.9	4799.9	3327.8	998.0	464.7
hsa-let-7d-5p	MIMAT0000065	6419.2	5729.5	4334.5	6996.3	7653.0	8463.9	5703.0	8485.1	7587.0	7381.3	9765.3	17322.6	14850.8	4868.8
hsa-let-7e-5p	MIMAT0000066	1135.1	12220.7	940.4	6093.1	4536.5	2901.6	27.4	2320.5	203.8	87.5	783.8	12.4	207.4	175.0
hsa-let-7f-5p	MIMAT0000067	28077.8	31777.1	8165.9	17125.9	29276.5	19973.1	9935.5	19680.1	105722.7	31636.4	7414.8	10400.3	33481.6	51633.5
hsa-let-7g-5p	MIMAT0000414	8550.7	6416.1	5405.9	6606.7	8709.1	8111.8	7245.7	8316.0	121519.6	25982.6	22060.0	23165.7	22722.5	21268.4
hsa-let-7i-5p	MIMAT0000415	8767.2	13644.8	8980.2	21659.4	18630.5	15206.3	11782.9	15445.5	25622.5	12732.9	42426.7	30302.2	10657.1	10663.7
hsa-miR-1-3p	MIMAT0000416	288.2	153.7	678.5	319.9	61.9	968.3	1539.6	218.1	74.5	346.4	7.6	1894.7	138.6	348.8
hsa-miR-100-5p	MIMAT0000098	1695.1	20479.4	6424.4	5349.3	13293.7	9368.7	5170.4	3067.4	192.2	2849.6	531.9	1576.6	11035.3	1276.0
hsa-miR-103a-3p	MIMAT0000101	2316.2	789.5	1370.3	3897.2	1116.2	3332.8	3476.3	2741.9	2594.7	1123.3	9689.5	4620.3	963.6	1507.8
hsa-miR-10a-5p	MIMAT0000253	3666.6	27949.7	6109.7	3047.1	9902.0	13208.4	7132.7	2501.0	198.0	173.8	1130.6	828.6	3163.6	522.7
hsa-miR-122-5p	MIMAT0000421	45289.8	516.1	55547.5	319.9	6919.5	6690.0	4346.4	4788.4	124.6	2633.8	3602.2	2717.5	19938.2	3478.1
hsa-miR-1249-3p	MIMAT0005901	504.6	147.4	226.9	461.6	480.1	1205.7	242.1	505.9	39.7	130.6	2169.1	2154.2	35.5	117.0
hsa-miR-125a-5p	MIMAT0000443	2565.5	5061.9	2266.3	5012.8	2991.7	2434.7	2421.2	3323.2	323.4	303.3	1605.3	1689.0	619.9	812.4
hsa-miR-125b-5p	MIMAT0000423	1408.0	6206.3	4464.3	4127.4	3153.4	3309.4	3372.6	14521.7	182.5	1209.6	1548.8	1634.8	3713.6	580.6
hsa-miR-126-3p	MIMAT0000445	21598.6	6784.8	9588.0	621.0	7023.1	3131.1	2828.5	3064.0	37417.1	24601.5	5921.0	1312.5	26022.4	18486.8
hsa-miR-127-3p	MIMAT0000446	7350.9	20358.6	8350.9	82260.1	49057.5	25435.2	17224.4	30016.3	2847.5	57617.8	1205.7	5.2	11344.6	2782.7
hsa-miR-1307-3p	MIMAT0005951	697.5	471.6	1118.1	1966.9	967.0	1149.9	1202.2	1055.2	487.4	260.1	2090.7	4295.6	860.5	175.0
hsa-miR-139-5p	MIMAT0000250	1968.0	605.1	1048.5	178.2	1024.2	1263.6	1169.4	1609.1	1402.1	5352.8	2183.8	1688.6	25094.3	4810.9
hsa-miR-143-3p	MIMAT0000435	8767.2	6638.6	5756.6	1205.4	1094.8	948.3	1254.4	657.8	582.0	303.3	2629.4	2070.9	8010.3	232.9
hsa-miR-146a-5p	MIMAT0000449	3657.2	4642.3	2254.3	7757.7	6247.6	5165.7	2908.8	4931.2	25958.3	7251.8	2091.5	2200.1	4401.0	6085.8
hsa-miR-149-5p	MIMAT0000450	749.3	1794.0	1653.8	2887.7	1982.9	1738.3	1551.1	2198.3	120.8	1036.9	2609.6	2981.8	6291.6	1334.0
hsa-miR-150-5p	MIMAT0000451	15764.0	2137.3	4882.2	2144.0	5126.2	11390.0	7149.6	8240.7	64757.9	27191.0	19599.2	18110.1	9969.7	19240.2
hsa-miR-151a-5p	MIMAT0004697	1742.1	3764.9	849.1	1347.0	1031.3	827.9	1212.6	347.2	6137.8	5180.2	1323.5	4.0	4022.9	3420.1
hsa-miR-155-5p	MIMAT0000646	565.8	1997.5	798.6	2108.5	1289.6	1424.1	324.5	961.6	4516.8	1598.0	1065.2	2594.3	757.4	36045.3
hsa-miR-15a-5p	MIMAT0000068	255.2	121.9	472.0	54.3	332.6	121.5	135.1	270.7	825.1	303.3	351.1	1290.7	138.6	348.8
hsa-miR-15b-5p	MIMAT0000417	1233.9	541.6	1202.2	1010.6	797.2	2024.6	1101.3	1142.0	4954.8	1641.2	1821.6	1604.1	860.5	1276.0
hsa-miR-16-5p	MIMAT0000069	16262.7	6072.8	9131.6	4676.4	8425.9	7943.6	5346.5	19177.6	27641.0	23997.3	13269.7	21690.8	13304.0	9562.7
hsa-miR-181a-5p	MIMAT0000256	38622.4	37384.6	46899.9	47674.2	34659.8	59005.5	48244.7	46861.9	54427.9	52740.9	181180.1	151561.2	47575.0	35581.7
hsa-miR-181b-5p	MIMAT0000257	5021.7	6009.2	5992.0	10874.6	5509.5	7197.0	7362.1	6471.7	5335.0	4230.7	19837.8	18473.0	12513.3	2666.8
hsa-miR-185-5p	MIMAT0000455	890.4	83.8	560.8	89.7	247.7	1112.1	606.2	2126.4	796.2	432.7	1223.3	1576.8	413.6	232.9
hsa-miR-191-5p	MIMAT0000440	7854.3	4979.2	8999.4	7137.9	5204.9	8330.2	7966.4	8069.4	19065.2	12603.4	18059.0	24612.9	14507.1	8519.6
hsa-miR-193a-5p	MIMAT0004614	95.3	312.7	452.7	2391.9	1490.6	558.3	656.6	430.5	35.9	44.3	986.1	3699.4	35.5	59.1
hsa-miR-196b-5p	MIMAT0001080	330.5	662.3	447.9	426.2	354.1	236.3	677.8	174.7	377.4	260.1	586.9	894.9	207.4	175.0
hsa-miR-197-3p	MIMAT0000227	706.9	579.7	1161.4	390.7	2565.5	869.2	1844.3	5083.0	475.9	648.5	6307.7	6515.3	551.1	522.7
hsa-miR-199a-3p	MIMAT0000232	20154.0	21414.0	8197.1	7970.3	17506.5	16835.3	5591.0	11069.4	10462.3	6345.5	11332.8	15199.8	3301.1	44505.8
hsa-miR-199b-3p	MIMAT0004563	10079.9	10701.2	4101.5	3985.7	8753.8	8418.2	2796.1	5535.3	5242.4	3194.9	5665.9	7600.5	1651.1	22253.5
hsa-miR-204-5p	MIMAT0000265	979.9	2328.1	443.1	249.1	480.1	327.6	648.3	125.6	53.2	475.9	29.5	2284.0	7494.7	24861.2
hsa-miR-21-5p	MIMAT0000076	36683.8	87400.9	15146.4	23324.1	16188.6	7478.9	6596.4	10455.0	64767.5	41044.9	11959.5	11079.1	21381.9	77420.8
hsa-miR-22-3p	MIMAT0000077	11317.4	6803.9	5634.1	2037.7	2467.2	3484.3	1946.5	2900.7	3696.6	23695.2	5624.6	4788.7	20591.3	333091.0
hsa-miR-221-3p	MIMAT0000278	1417.4	2181.8	863.5	11795.4	1471.8	756.6	745.2	342.6	2035.1	1598.0	2757.3	1612.3	1341.7	986.3
hsa-miR-222-3p	MIMAT0000279	6480.4	14147.1	5773.4	67419.8	26264.5	4853.7	11204.6	8859.6	1627.9	993.8	978.9	3166.3	5672.9	1102.2
hsa-miR-223-3p	MIMAT0000280	3930.1	605.1	1151.8	443.9	2502.9	1292.6	1355.9	1924.3	26253.5	11783.4	3377.9	4427.8	5776.0	7013.0
hsa-miR-23a-3p	MIMAT0000078	6706.2	12951.8	4783.7	8289.0	7572.6	8400.4	5572.6	6467.1	22322.6	10531.8	11721.2	9163.4	7735.3	8635.5
hsa-miR-23b-3p	MIMAT0000418	1144.5	1088.3	640.1	72.0	237.9	431.2	256.5	19.4	2259.0	648.5	674.9	664.4	688.6	1102.2
hsa-miR-24-3p	MIMAT0000080	16709.7	18057.1	25213.7	19251.0	16459.3	20199.3	14589.4	15524.3	24858.3	17523.5	45282.8	53489.2	17532.0	12286.3
hsa-miR-25-3p	MIMAT0000081	1012.8	732.3	1317.5	1701.2	1637.1	609.5	905.4	1352.1	2982.6	950.6	2785.4	2615.2	3816.7	638.6
hsa-miR-26a-5p	MIMAT0000082	40749.2	39953.1	14889.4	13708.0	14638.4	10542.0	13273.9	11473.7	112982.4	77945.5	28227.5	26250.9	35578.4	50648.4
hsa-miR-26b-5p	MIMAT0000083	2932.6	2582.4	753.0	1293.9	1775.6	1823.0	685.7	733.2	23453.4	8546.5	1967.5	521.9	2613.6	3883.7
hsa-miR-27a-3p	MIMAT0000084	5172.3	2728.6	2741.9	1701.2	2083.9	2636.4	3046.0	1674.2	13573.1	7036.0	5823.6	6933.1	2648.0	5158.6
hsa-miR-27b-3p	MIMAT0000419	2495.0	1183.7	995.6	213.7	949.1	962.7	998.0	1190.0	3021.2	2935.9	1305.2	3133.6	1823.0	1334.0
hsa-miR-29a-3p	MIMAT0000086	725.8	910.3	584.9	691.8	125.3	692.0	507.1	331.2	3304.9	907.5	563.9	255.7	448.0	812.4
hsa-miR-30d-5p	MIMAT0000245	2297.3	2391.7	2064.6	868.9	1520.1	2240.8	2100.3	3278.7	7878.4	3669.6	4158.8	909.3	3301.1	3362.2
hsa-miR-320a-3p	MIMAT0000510	5106.4	5061.9	7125.8	28743.1	11980.2	7795.4	8351.0	10351.1	2056.3	35175.4	14461.6	19816.5	1857.4	45433.0
hsa-miR-320c	MIMAT0005793	245.8	274.5	584.9	1293.9	297.8	414.5	1020.3	475.1	57.1	44.3	27.4	1192.9	241.8	348.8
hsa-miR-328-3p	MIMAT0000752	1229.2	630.6	1310.3	1081.4	1281.5	1659.2	1572.7	1534.8	113.1	1080.1	4023.7	2477.8	1101.1	1160.1
hsa-miR-342-3p	MIMAT0000753	2029.1	795.9	558.4	922.0	675.7	583.9	741.9	561.9	10618.6	4662.3	1017.7	2870.8	5260.4	2435.0
hsa-miR-345-5p	MIMAT0000772	307.0	312.7	1161.4	638.7	222.7	1161.1	2080.9	862.2	255.9	173.8	5287.1	745.6	104.3	290.9
hsa-miR-34a-5p	MIMAT0000255	6936.8	18336.8	7572.6	11476.7	12203.6	11298.6	2931.5	5980.6	446.9	5050.7	1017.4	2124.7	14678.9	10779.6
hsa-miR-369-5p	MIMAT0001621	1116.3	3625.1	2167.8	1506.4	2730.8	3746.2	2147.1	2216.6	400.6	821.2	4.7	632.4	1169.9	348.8
hsa-miR-378a-3p	MIMAT0000732	3986.5	3542.4	5192.1	2374.2	3916.4	8655.6	4833.6	4402.4	1423.4	9323.4	20153.7	15699.4	688.6	232.9
hsa-miR-411-5p	MIMAT0003329	862.2	2226.4	798.6	1453.3	1269.0	539.3	267.6	402.0	298.3	216.9	906.3	3.1	276.1	175.0
hsa-miR-423-3p	MIMAT0001340	10781.0	10542.3	16734.2	23536.6	16068.0	23946.5	14662.9	24952.7	4676.9	6129.7	88777.0	74259.4	18047.6	4868.8
hsa-miR-423-5p	MIMAT0004748	2504.4	1342.6	2818.8	2427.3	4592.8	4357.9	4113.4	8607.2	483.6	44.3	3777.9	10811.5	413.6	59.1
hsa-miR-424-5p	MIMAT0001341	556.4	331.7	719.4	355.3	573.0	396.7	712.4	249.0	186.4	44.3	31.7	2.9	69.9	59.1
hsa-miR-425-3p	MIMAT0001343	227.0	39.3	164.5	319.9	116.4	696.4	237.4	383.7	881.1	346.4	261.3	815.2	379.3	117.0
hsa-miR-425-5p	MIMAT0003393	1083.4	598.8	659.3	231.4	128.0	449.1	560.8	622.4	2347.7	1598.0	3057.7	2616.3	1032.4	870.4
hsa-miR-451a	MIMAT0001631	100356.2	17268.7	20995.6	9263.0	78801.1	56569.8	41488.8	38475.1	5595.5	13552.9	46456.4	48226.4	69952.6	854978.0
hsa-miR-484	MIMAT0002174	838.7	821.3	2136.6	2834.6	1506.7	2736.6	2626.8	2193.8	499.0	346.4	9382.2	9660.7	344.9	348.8
hsa-miR-485-5p	MIMAT0002175	688.1	433.5	2557.0	1400.2	3382.1	3661.5	3009.3	1964.2	47.5	5180.2	1037.9	1.4	310.5	117.0
hsa-miR-486-5p	MIMAT0002177	4240.6	541.6	4128.0	1.1	8772.6	6851.6	6085.4	6030.9	651.5	2159.1	5237.2	2404.2	2166.7	1276.0
hsa-miR-532-5p	MIMAT0002888	951.6	1056.5	611.3	532.4	559.6	707.6	1243.9	544.7	529.9	216.9	1349.1	793.0	1101.1	175.0
hsa-miR-574-3p	MIMAT0003239	1008.1	961.2	1245.4	975.1	1285.1	456.9	672.1	1042.6	271.3	389.6	2493.2	572.7	35.5	9504.8
hsa-miR-652-3p	MIMAT0003322	5384.0	3421.6	5732.6	5012.8	7909.5	9459.0	10485.1	6726.4	2488.6	4230.7	22102.4	20169.3	3541.7	1276.0
hsa-miR-7-5p	MIMAT0000252	3374.9	7903.8	1968.5	14558.1	11374.4	5586.9	1344.0	6094.8	8698.5	103495.3	956.6	2720.8	13304.0	33205.8
hsa-miR-744-5p	MIMAT0004945	711.6	675.1	1396.8	1028.3	1654.1	1806.2	2784.9	1363.5	230.8	130.6	6549.5	3713.4	723.0	117.0
hsa-miR-9-5p	MIMAT0000441	523.4	12360.6	123.7	957.4	191.5	34.6	755.2	1607.9	441.1	15538.2	4127.9	10.0	16397.6	1565.8
hsa-miR-92a-3p	MIMAT0000092	176215.3	62148.0	161663.1	89662.5	176098.1	203969.3	196439.2	169790.1	23532.6	27967.9	275499.6	221993.4	103192.5	99151.6
hsa-miR-92b-3p	MIMAT0003218	8451.9	30435.6	48079.3	59202.8	77674.4	93813.8	89624.7	59864.7	414.1	6129.7	852.4	823.5	38740.9	16400.7
hsa-miR-93-5p	MIMAT0000093	3003.1	1139.2	3095.1	3029.4	2374.3	3009.6	1936.4	2608.3	5981.4	2461.2	11095.6	9557.4	4263.5	3536.0
hsa-miR-99a-5p	MIMAT0000097	6438.0	4025.6	3565.9	603.3	1004.5	848.0	1699.8	865.6	489.4	3497.0	2014.9	3264.6	18597.6	14198.6
hsa-miR-99b-5p	MIMAT0000689	2570.2	6225.4	3674.0	3808.6	4088.0	2283.1	3882.9	2207.5	541.5	1339.1	1502.9	16.5	3369.8	1507.8

Table S2. miRNAs matched to the coding region binding sites of SARS-CoV-2.

miRNA	Expression (%)[1]	Score[2]
hsa-miR-181a-5p	4.69	70
hsa-miR-26a-5p	1.58	68
hsa-miR-199a-3p	1.31	69
hsa-miR-320a-3p	1.15	73
hsa-miR-16-5p	0.78	99
hsa-miR-23a-3p	0.74	79
hsa-miR-181b-5p	0.73	70
hsa-miR-199b-3p	0.65	69
hsa-miR-122-5p	0.64	67
hsa-miR-378a-3p	0.49	55
hsa-miR-27a-3p	0.29	67
hsa-miR-103a-3p	0.26	85
hsa-miR-197-3p	0.25	82
hsa-miR-30d-5p	0.20	95
hsa-miR-26b-5p	0.14	68
hsa-miR-139-5p	0.12	83
hsa-miR-27b-3p	0.11	67
hsa-miR-15b-5p	0.11	99
hsa-miR-532-5p	0.10	61
hsa-miR-155-5p	0.09	51
hsa-miR-1-3p	0.09	54
hsa-miR-411-5p	0.09	88
hsa-miR-320c	0.07	73
hsa-miR-424-5p	0.07	98
hsa-miR-196b-5p	0.06	52
hsa-miR-29a-3p	0.04	91
hsa-miR-23b-3p	0.03	79

1 indicates "Percentage of miRNAs in EVs".
2 indicates the prediction score or the miRDB method. According to miRDB, a predicted target with score > 80 is most likely to be real.
